# Single-Ended Eddy Current Micro-Displacement Sensor with High Precision Based on Temperature Compensation

**DOI:** 10.3390/mi15030366

**Published:** 2024-03-07

**Authors:** Zhengping Xu, Yongtong Feng, Yi Liu, Fengxin Shi, Yang Ge, Han Liu, Wei Cao, Hong Zhou, Shuang Geng, Wenqi Lin

**Affiliations:** 1 Suzhou Institute of Biomedical Engineering and Technology, Chinese Academy of Sciences, Suzhou 215163, China; fengyt@sibet.ac.cn (Y.F.); liuyi@sibet.ac.cn (Y.L.); shifengxin@sibet.ac.cn (F.S.); gey@sibet.ac.cn (Y.G.); caow@sibet.ac.cn (W.C.); zhouh@sibet.ac.cn (H.Z.); linwq@sibet.ac.cn (W.L.); 2 School of Biomedical Engineering (Suzhou), Division of Life Sciences and Medicine, University of Science and Technology of China, Hefei 230026, China; 3 Suzhou Guoke Medical Technology Development (Group) Co., Ltd., Suzhou 215163, China; liuhan@sibet.ac.cn (H.L.); gengshuang@sibet.ac.cn (S.G.)

**Keywords:** eddy current sensor, micro-displacement, digital phase discriminator, temperature compensation

## Abstract

To measure the micro-displacement reliably with high precision, a single-ended eddy current sensor based on temperature compensation was studied in detail. At first, the principle of the eddy current sensor was introduced, and the manufacturing method of the probe was given. The overall design plan for the processing circuit was induced by analyzing the characteristics of the probe output signal. The variation in the probe output signal was converted to pulses with different widths, and then it was introduced to the digital phase discriminator along with a reference signal. The output from the digital phase discriminator was processed by a low-pass filter to obtain the DC component. At last, the signal was amplified and compensated to reduce the influence of temperature. The selection criteria of the frequency of the exciting signal and the design of the signal conditioning circuit were described in detail, as well as the design of the temperature-compensating circuit based on the digital potentiometer with an embedded temperature sensor. Finally, an experimental setup was constructed to test the sensor, and the results were given. The results show that nonlinearity exists in the single-ended eddy current sensor with a large range. When the range is 500 μm, the resolution can reach 46 nm, and the repeatability error is ±0.70% FR. Within the temperature range from +2 °C to +58 °C, the voltage fluctuation in the sensor is reduced to 44 mV after temperature compensation compared to the value of 586 mV before compensation. The proposed plan is verified to be feasible, and the measuring range, precision, and target material should be considered in real-world applications.

## 1. Introduction

With the continuous development of semiconductor technology, precision manufacturing technology, micro-electromechanical technology, and nanotechnology, there is an increasing demand for the high-precision measurement of various displacements and vibrations. Additionally, in applications such as angle measurements of fast steering mirrors, research on mechanical vibration technology, and continuous monitoring processes, non-contact measurements are required. Therefore, non-contact, high-precision micro-displacement sensors are key to meeting the aforementioned requirements.

Common non-contact sensors include optical (including laser and fiber optic) interferometric [1], capacitive [2,3,4,5], and eddy current [6,7,8] sensors. Optical interferometric displacement sensors are irreplaceable in specific applications due to their advantages, such as immunity to electromagnetic interference, large measurement distance and range, and lack of requirements on the target material. However, they are expensive and complex in structure, making miniaturization difficult. With the continuous development of light sources and optical systems, optical interferometric displacement sensors have made significant progress in terms of price and size, but they still lack advantages compared to capacitive and eddy current sensors in terms of these parameters. Capacitive sensors are currently the most accurate non-contact sensors, with advantages such as small size, high resolution, bandwidth, linearity, and stability, and are widely used in nano-positioning platforms, acceleration sensors, and pressure sensors. However, capacitive sensors are susceptible to influence from media, the environment, and contaminants, thus limiting their application in sealed enclosures or environments with strict control measures. Eddy current sensors, based on the electromagnetic induction principle, convert displacement [9], amplitude, thickness, size, cracks, and other information of the measured target into measurements of Q value, the equivalent impedance Z, and the equivalent inductance L of the sensor coil [10]. They have advantages such as a simple structure, low price, high sensitivity, wide frequency band, resistance to influence from oily media, and strong anti-interference ability. They are widely used in measurements of displacement, vibration, rotation speed, thickness of metal films, geometric dimensions of metal components, surface roughness, temperature, and other physical quantities [11,12,13,14,15,16,17]. They play important roles in industrial production, aerospace manufacturing, the nuclear industry, the petrochemical industry, safety testing, etc. In the field of mechanical vibration technology, eddy current sensors are mainly used for radial vibration measurements of rotating shafts and axial displacement measurements; in optoelectronic imaging systems, they are mostly used for angle measurements of fast steering mirrors [18,19]. According to the application method, eddy current sensors are divided into single-ended and differential types. In situations where installation space allows for it, the differential type achieves better linearity within a large measurement range [20].

Temperature drift is one of the key issues that need to be addressed in the design and application of eddy current sensors, and the handling methods mainly include data processing and additional temperature compensation modules. Lei et al. proposed a temperature compensation method based on binary regression, reducing the maximum relative measurement error from 169.08% to 9.13% [21]. Li and Ding utilized a non-inductive compensating coil to design a displacement eddy current sensor, reducing the temperature drift from 12% to 0.7% within a temperature range from 20 °C to 90 °C [22]. Wang et al. designed a displacement sensor based on a constant-current circuit and a temperature coefficient parameter method, achieving a temperature drift rate of ±0.96 μm/°C within a temperature range from 40 °C to 55 °C [23].

This paper constructs a temperature compensation module based on a digital potentiometer with an embedded temperature sensor, completing the design of a high-precision single-ended eddy current micro-displacement sensor. The core of the signal conditioning circuit is the change in voltage amplitude and phase caused by the change in the equivalent impedance of the sensor probe. Using the reference signal as a standard, the variation is converted into different pulse widths. Subsequently, the signal undergoes processing through a low-pass filter, voltage amplifier, and temperature compensation stages to derive the final measurement result. This paper elaborates on the selection criteria of the frequency of the exciting signal, the design of the signal conditioning circuit, the signal amplification, and the design of the temperature compensation circuit. In conclusion, an experimental setup is constructed for testing the sensor, and the experimental results are presented.

## 2. Principle of Eddy Current Sensor

The probe in the eddy current sensor is a flat coil fixed on a framework, forming a resonant circuit with a capacitor during operation. An exciting signal with a frequency exceeding 500 kHz is applied to the probe coil to generate a magnetic field. When the sensor probe approaches a measured target, such as a metal conductor, eddy currents are induced on its surface by the magnetic field. These eddy currents, intersecting with the exciting magnetic field, create a time-varying magnetic field that impedes the variation of the exciting magnetic field. From an energy perspective, the conductor experiences both eddy current losses and magnetic losses. Due to the high frequency of the exciting signal, the eddy current losses are much greater than the magnetic losses. Energy losses cause changes in the Q value, equivalent impedance Z, and equivalent inductance L of the sensor coil. Therefore, by measuring these electrical quantities, the distance between the measured conductor and the sensor probe can be determined.

**Figure 1:**
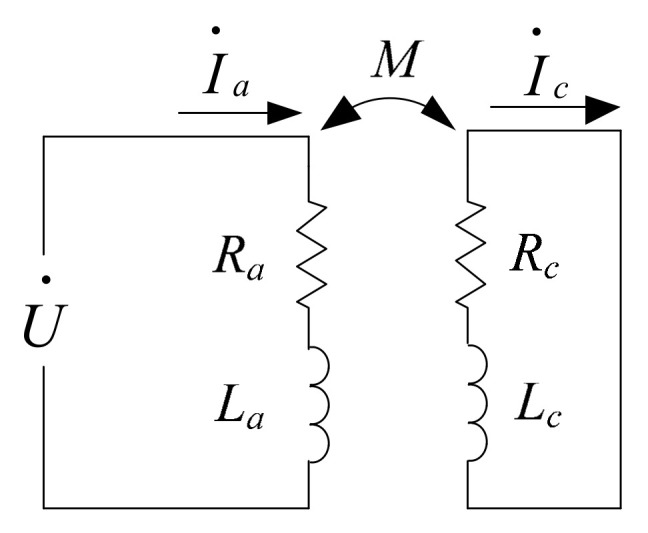
Principle diagram of the interaction between the measured conductor and sensor probe.

The principle diagram of the principle is shown in Figure 1.

In Figure 1, U represents the exciting voltage across the terminals of the eddy current sensor coil; *I_a_* and *I_c_*, respectively, represent the exciting current and eddy current; *R_a_* and *L_a_* are the resistance and inductance of the sensor coil when the eddy current effect is not considered; *R_c_* and *L_c_* are the equivalent resistance and inductance of the eddy current in the measured conductor; and *M* represents the equivalent mutual inductance between the coil and the eddy current loop.

According to Figure 1, the following system of equations can be derived:

(1) $$\left[\begin{array}{cc} R_{a}+j\omega L_{a} & -j\omega M\\ -j\omega M & R_{c}+j\omega L_{c} \end{array}\right]\left[\begin{array}{c} {\overset{\cdot }{I}}_{a}\\ {\overset{\cdot }{I}}_{c} \end{array}\right]=\left[\begin{array}{c} \overset{\cdot }{U}\\ 0 \end{array}\right]$$

In the equations, *ω* represents the angular frequency of the exciting signal, measured in radians per second (rad/s).

Solving the above system of equations yields the expression for the equivalent impedance of the eddy current sensor coil after introducing the measured conductor as follows:

(2) $$\left\{\begin{array}{c} \begin{array}{l} {\overset{\cdot }{Z}}_{a}=\left[R_{a}+\frac{\omega^{2}M^{2}}{R_{c}^{2}+{\left(\omega L_{c}\right)}^{2}}R_{c}\right]+j\omega L\\ L=L_{a}-\frac{\omega^{2}M^{2}}{R_{c}^{2}+{\left(\omega L_{c}\right)}^{2}}L_{c} \end{array}\\ M^{2}=K_{M}\left(1-pd^{2}+qd^{4}-\cdots +\cdots \right) \end{array}\right.$$

In the equations, *K_M_* is the mutual inductance coefficient; *p* and *q* are coefficients related to the coil parameters; and *d* is the distance between the measured conductor and the sensor.

Generally, the value of *R_c_* is very small. In the case of a high exciting signal angular frequency, the expression for the equivalent impedance of the eddy current sensor coil after introducing the measured conductor can be simplified to the following form as follows:

(3) $${\overset{\cdot }{Z}}_{a}\approx R_{a}+j\omega L_{a}-jK_{M}\omega \frac{1-pd^{2}+qd^{4}-\cdots +\cdots }{L_{c}}$$

**Figure 2:**
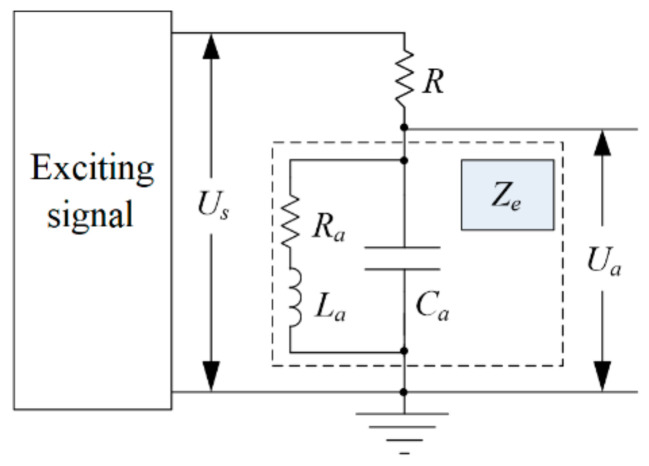
Principle diagram of the measuring circuit of eddy current sensor.

Once the design of the eddy current sensor is completed, *R_a_*, *L_a_*, and *ω* are all fixed values. It can be observed that the equivalent impedance of the eddy current sensor coil is dependent on the distance *d* between the measured conductor and the eddy current sensor. By connecting the sensor probe in parallel with a capacitor, let its equivalent impedance be *Z_e_*. Through resistor *R*, a stable high-frequency signal is applied to the above parallel circuit as the exciting signal. Then, the resistor and the parallel circuit form a voltage divider circuit. The principle diagram is illustrated in Figure 2.

Assuming that the amplitude of the exciting signal is *U_s_*, the calculation expression for the voltage *U_a_* across the parallel circuit is as follows:

(4) $${\overset{\cdot }{U}}_{a}=\frac{Z_{e}}{R+Z_{e}}\times {\overset{\cdot }{U}}_{s}$$

From the above expression, it can be seen that the relationship between the equivalent impedance of the eddy current sensor coil and the distance between the measured conductor and the eddy current sensor probe is complex, exhibiting significant nonlinear characteristics. The signal obtained, according to Figure 2, is an AC signal. Further conditioning is required to obtain a DC signal corresponding to displacement, facilitating further processing.

## 3. Design of the Probe

The core of the probe is the coil, and its main technical parameters include the inner diameter, outer diameter, number of windings, coil thickness, inductance, and resistance, among others [24]. The research indicates that the measurement range is directly correlated with the outer diameter of the coil; the larger the outer diameter, the wider the measurement range. Meanwhile, the inductance and resistance determine the selection of parameters for subsequent signal conditioning circuits.

**Figure 3:**
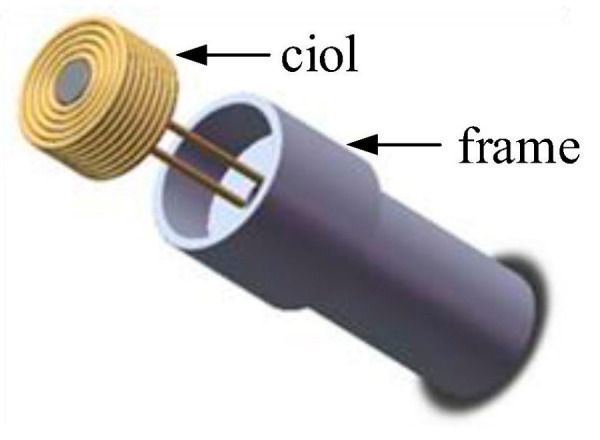
Structure diagram of probe.

While designing, reliable fixation of the coil and facilitating assembly during application should be ensured. A structure diagram of the probe is shown in Figure 3.

**Figure 4:**
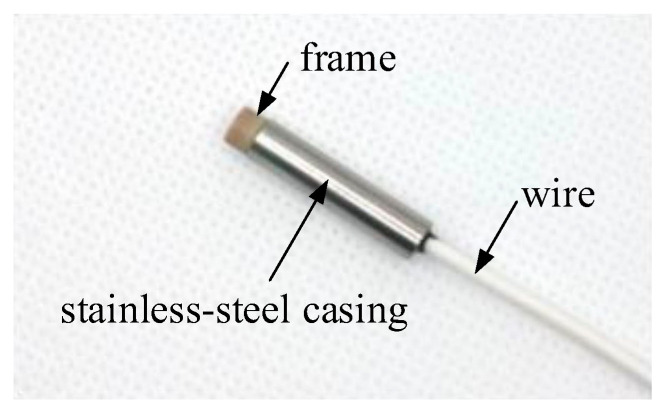
Physical illustration of the designed probe.

The coil, wound with high-strength enameled copper wire in multiple layers and turns, is initially secured to the frame using adhesive. PEEK material is selected for the frame. Subsequently, the frame is inserted into the stainless-steel casing. It is imperative to ensure the flatness of the coil’s end face during the manufacturing process. The physical illustration of the designed probe is shown in Figure 4.

## 4. Design of Signal Conditioning Circuit

### 4.1. Characteristics of Probe Output Signal

**Figure 5:**
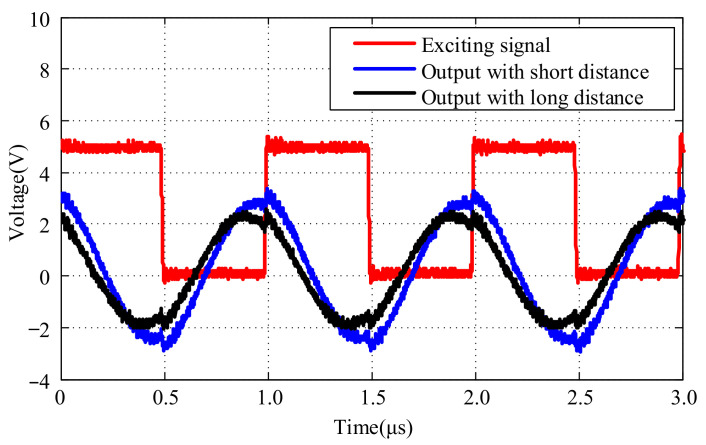
Probe signals according to measured targets with different distances.

The foundation for designing the signal conditioning circuit is to analyze the correlation between the output signal of the sensor probe and its distance from the measured target. Output signals corresponding to different distances between the sensor probe and the measured target were collected, as shown in Figure 5.

From Figure 5, it is evident that the closer the distance to the target being measured, the larger the amplitude of the output signal from the sensor probe, and vice versa. By setting the same reference voltage, the probe output signal is converted into a corresponding square wave signal. It can be observed that when the measured target is at different distances, the phase difference between the square wave signal of the probe and the exciting signal varies. This observation forms the basis for the subsequent design of the signal conditioning circuit.

### 4.2. Overall Circuit Design

**Figure 6:**
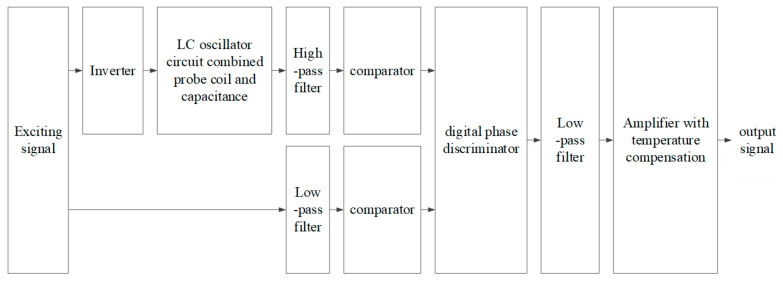
Block diagram of signal conditioning circuit of the single-end sensor with temperature compensation.

The core concept of the single-ended micro-displacement measurement circuit with temperature compensation is to convert the phase variation of the sensor probe signal caused by the distance change between the probe and the measured target into signals with different pulse widths. These signals are then compared with a reference signal to obtain the variation of the pulse width. Subsequently, the DC component is obtained through a low-pass filter, followed by a voltage amplifier and temperature compensation to get the desired result. The block diagram of the signal conditioning circuit is illustrated in Figure 6. The key points include the selection of exciting signals, the design of the low-pass filter, and the signal amplifier with temperature compensation.

### 4.3. Frequency Selection of Exciting Signal

The frequency selection of the exciting signal is related to the resonant frequency *f*_0_ of the *LC* oscillation circuit formed by the probe coil and capacitor in parallel. When the frequency of the exciting signal matches *f*_0_, the output signal is maximum. During measurement, as the sensor probe approaches or moves away from the measured target, the equivalent impedance of the *LC* oscillation circuit changes, causing the circuit to become detuned. Therefore, the exciting signal frequency should be chosen near *f*_0_. The expression for the resonant frequency *f*_0_ of the *LC* oscillation circuit is as follows:

(5) $$f_{0}=\frac{1}{2\pi \sqrt{LC}}$$

In the equation, *L* represents the intrinsic inductance of the sensor probe, measured in Henrys (H). *C* represents the parallel capacitance, measured in Farads (F).

According to the system design, the resonant frequency of the LC oscillation circuit formed by the sensor probe and the parallel capacitor is 1.2 MHz. An exciting signal with a frequency of 1 MHz is selected.

### 4.4. Design of Signal Conditioning Circuit

**Figure 7:**
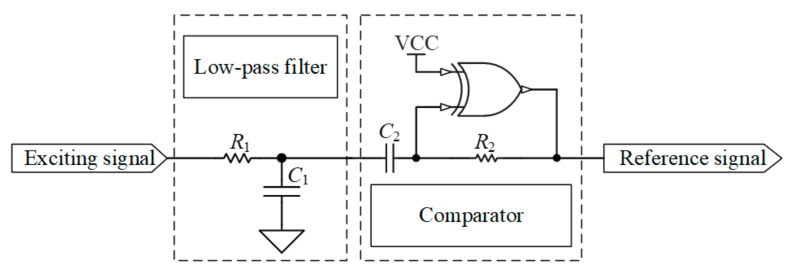
Circuit diagram of low-pass filter and comparator.

The low-pass filter connected with the exciting signal is implemented using an RC circuit, as shown in Figure 7.

The time constant *τ* of the low-pass filter is determined by the values of the resistor *R*_1_ and the capacitor *C*_1_, and is expressed as follows:

(6) $$\tau =R_{1}C_{1}$$

**Figure 8:**
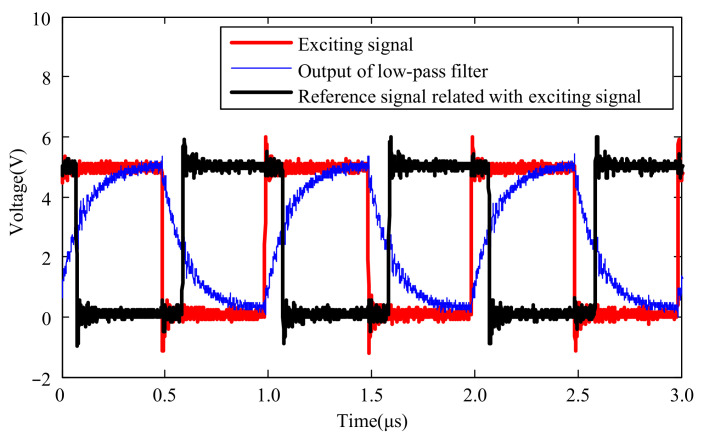
Reference generated by comparator and low-pass filter with exciting signal applied.

The selection of this time constant should ensure that the capacitor voltage approaches the charging maximum voltage or the discharging completion voltage as closely as possible within half the period of the exciting signal. Considering the period of the exciting signal is 1 μs, a time constant of 70 ns is chosen for the low-pass filter. After passing through the low-pass filter, the exciting signal is further processed by a comparator to generate a reference signal, as shown in Figure 7. The comparator consists of an XOR gate connected to a resistor and capacitor. One pin of the XOR gate is connected to VCC to form an inverter, and the comparison voltage of the comparator is determined by the values of resistor *R*_2_ and capacitor *C*_2_. The relevant curve is shown in Figure 8. 

It is evident that the exciting signal causes the RC circuit to undergo charging and discharging, forming a quasi-sawtooth-shaped waveform. This waveform is then processed by the comparator to generate the reference signal, which is subsequently phase-compared with the signal generated by the detector coil.

Since the quasi-sawtooth-shaped waveform signal corresponds to the charging state during the high level of the exciting signal, and as in Figure 3, the signal of the sensor probe corresponds to the rising phase when the applied exciting signal is low. Therefore, to ensure proper phase comparison with the reference signal, the exciting signal needs to be inverted before being applied to the sensor probe.

**Figure 9:**
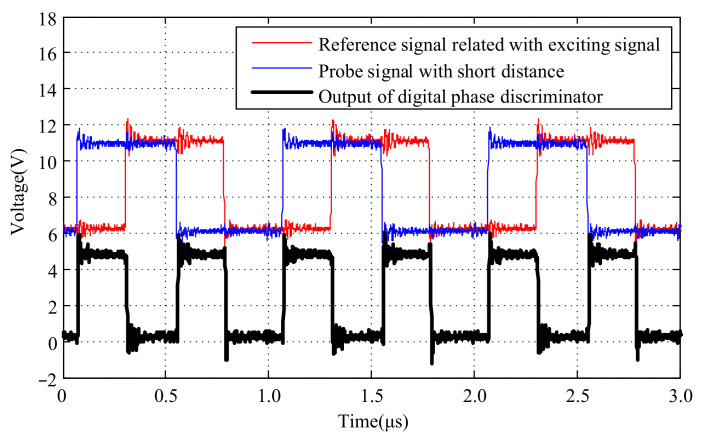
Output of digital phase discriminator according to target with short distance.

**Figure 10:**
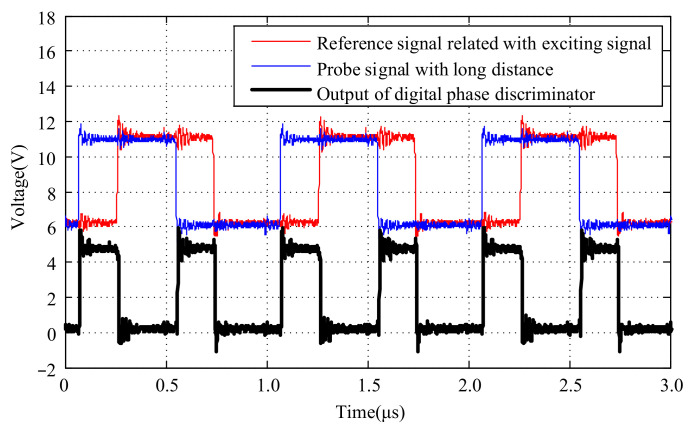
Output of digital phase discriminator according to target with long distance.

The phase relationships and amplitude information of the probe signal, the reference signal generated by the exciting signal, and the output signal of the digital phase discriminator at different distances are depicted in Figure 9 and Figure 10. To facilitate the observation of the phase relationships and amplitude information among those signals, a DC bias of 6.0 V was added to the probe signal and the reference signal generated by the exciting signal.

It can be observed that at different distances, the signals generated by the probe exhibit varying phases relative to the reference signals generated by the exciting signal. The phase difference is detected by a digital phase discriminator to get pulse signals with different pulse widths.

**Figure 11:**
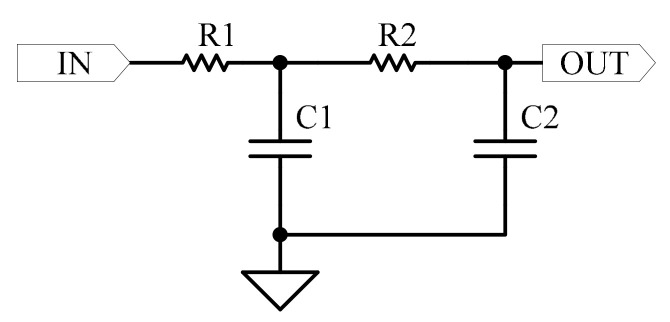
Block diagram of low-pass filter.

The output signal of the digital phase discriminator needs to be converted into a DC signal for subsequent processing. This functionality is realized by the use of a low-pass filter, as illustrated in Figure 11.

The transfer function expression corresponding to Figure 11 is as follows:

(7) $$G_{LP}\left(s\right)=\frac{u_{OUT}}{u_{IN}}=\frac{1}{R_{1}R_{2}C_{1}C_{2}s^{2}+(R_{1}C_{1}+R_{1}C_{2}+R_{2}C_{2})s+1}$$

When *R*_1_ *= R*_2_ *= R* and *C*_1_ *= C*_2_ *= C*, the transfer function expression above becomes:

(8) $$G_{LP}\left(s\right)=\frac{u_{OUT}}{u_{IN}}=\frac{\omega_{n}^{2}}{s^{2}+2\times 1.5\times \omega_{n}s+\omega_{n}^{2}}$$

**Figure 12:**
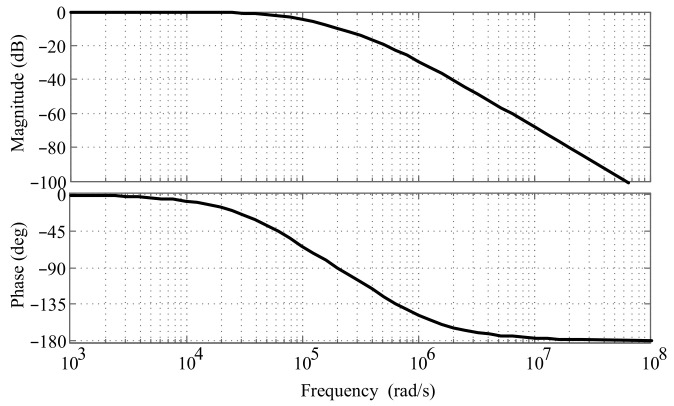
Bode diagram of transfer function of low-pass filter when *ω_n_* is 2 × 10^5^ rad/s.

The damping ratio of the circuit is 1.5, and the natural oscillation frequency *ω_n_* is determined by the RC value, with the relationship *ω_n_* = 1/*RC*. Taking the natural oscillation frequency *ω_n_* as 2 × 10^5^ rad/s, the corresponding Bode diagram of the transfer function is depicted in Figure 12.

The frequency of the output signal from the digital phase discriminator is 2 MHz, corresponding to an angular frequency of 12.5664 × 10^6^ rad/s. From the frequency response curve, it can be observed that the gain of the low-pass filter at this frequency is less than −40 dB, resulting in a DC signal output. The output signal of the low-pass filter is amplified and temperature-compensated to get the final result. The gain is set by a potentiometer and can be adjusted as needed.

### 4.5. Design of Signal Amplification and Temperature Compensation Circuit

**Figure 13:**
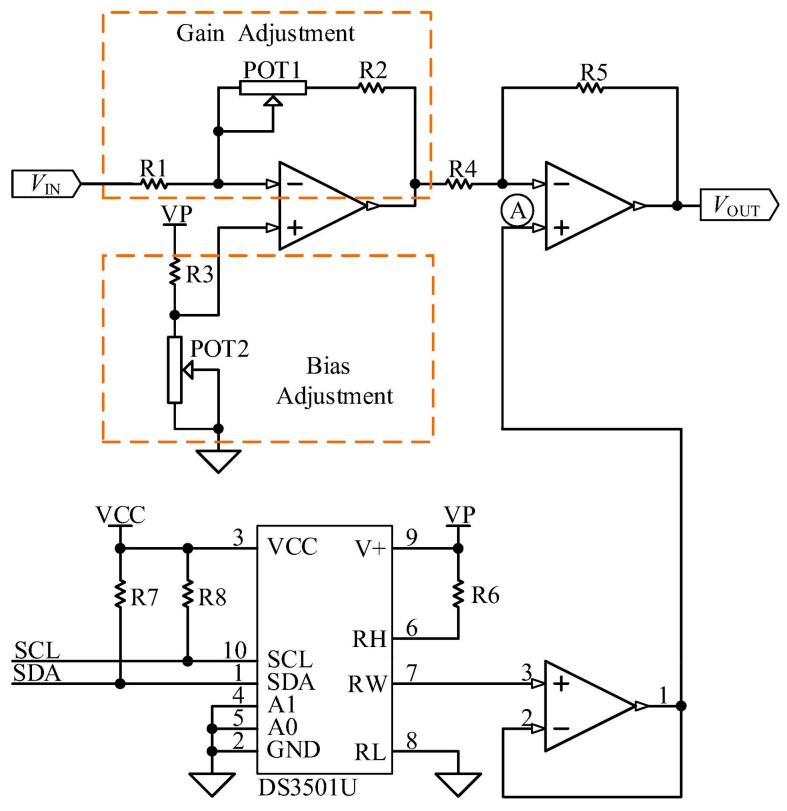
Circuit diagram of gain and bias adjustment and temperature compensation.

To facilitate the adjustment of sensitivity and output measuring range, the gain and bias adjustment circuits are induced in the system. Additionally, to improve the temperature characteristics of the sensor, compensation is achieved using a digital potentiometer with an embedded temperature sensor. The circuit diagram is shown in Figure 13.

The DS3501U [25] digital potentiometer from Maxim Integrated Products, San Jose, CA, USA, with an embedded temperature sensor is selected, and its terminal resistance is 10 kΩ. It features a 36-byte, 7-bit, non-volatile lookup table to store wiper values corresponding to different temperatures, covering a temperature range from −40 °C to +100 °C. Each 4 °C temperature interval corresponds to a specific wiper value to determine the position of the middle tap of the digital potentiometer. For example, register *LUT*[0] stores the wiper value used when the temperature is equal to or less than −37 °C, and *LUT*[1] stores the wiper value used when the temperature is between −36 °C and −33 °C, and so on. Programming and configuration can be achieved through the I^2^C bus. The DS3501U operates in three modes as follows: default mode, lookup table mode, and lookup table address mode. For temperature compensation, it typically operates in the lookup table mode, where the output resistance is directly determined by the data in the lookup table.

The relationship expression for the circuit shown in Figure 13 is as follows:

(9) $$V_{OUT}=V_{IN}\times \frac{\left(R_{POT1}+R_{2}\right)R_{5}}{R_{1}R_{4}}-VP\times \left[\frac{R_{5}R_{POT2}\left(R_{1}+R_{2}+R_{POT1}\right)}{R_{1}R_{4}\left(R_{3}+R_{POT2}\right)}-\frac{P\times R_{total}\left(R_{4}+R_{5}\right)}{127\times R_{4}\left(R_{6}+R_{total}\right)}\right]$$

where *R_total_* is the terminal resistance of the DS3501U, with a value of 10 kΩ. It can be observed that POT1 is used to set the circuit gain, while POT2 and the digital potentiometer DS3501U are used for bias adjustment. Note that potentiometer POT1 affects the output bias.

**Figure 14:**
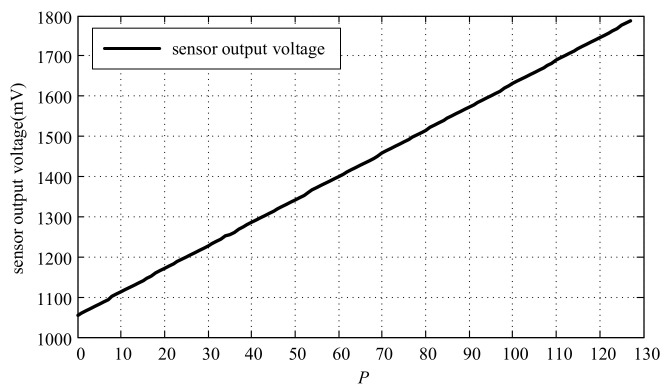
Relationship curve between sensor output voltage and wiper value *P*.

Regarding the setting sensor measurement range to 500 μm, when the measured target is fixed at a certain position, and the wiper value *P* is adjusted (with *P* ranging from 0 to 127), the relationship curve between the sensor output voltage and the wiper value *P* is obtained, as shown in Figure 14.

It is evident that the adjustment range of the output voltage can reach 730 mV, based on which temperature compensation is performed. Combined with the adjustment of the measurement range, the specific steps are as follows:

1. Firstly, under room temperature conditions, set all the wiper values *P* in the lookup table to 64;
2. Adjust the measured target to the desired maximum distance from the sensor probe, and adjust the potentiometer *POT2* to achieve the minimum expected output voltage of 0 V;
3. According to the measurement range, move the measured target to the minimum distance and adjust potentiometer *POT1* to achieve the maximum expected output voltage of 10 V;
4. Repeat steps 2–3 until the output reaches, respectively, the minimum output value (0 V) and maximum output value (10 V) when the measured target is furthest and closest to the sensor probe;
5. Adjust the distance between the probe and the measured target so that the output voltage is +1.95 V and +4.20 V, respectively. Then, place the probe, experimental setup, and processing circuit in a high-low temperature test chamber, with a temperature range from +2 °C to +58 °C. Record the corresponding output voltage every 4 °C and observe the trend of the output change with the temperature;
6. Calculate the voltage variation Δ*U*_max_ with the temperature range from +2 °C to +58 °C. Select the specified temperature output voltage as the basis and determine the corresponding wiper value register *LUT*[*k*] for this temperature. Calculate and update other wiper value registers according to the following formula:

(10) $$LUT\left[i\right]=64+\left(i-k\right)\times \frac{\Delta U_{\max}\times 128}{0.730\times 15}$$

In the above formula, the constant 15 is the number of value registers related to the temperature range from +2 °C to +58 °C. Considering the range of temperature compensation, i could be a value range from 10 to 24;

7. Adjust the distance between the probe and the measured target again so that the output voltage is +1.95 V. Conduct a temperature test according to step 5, record the data, and compare it with the results before temperature compensation.

## 5. Experiment

### 5.1. Circuit Design Results

**Figure 15:**
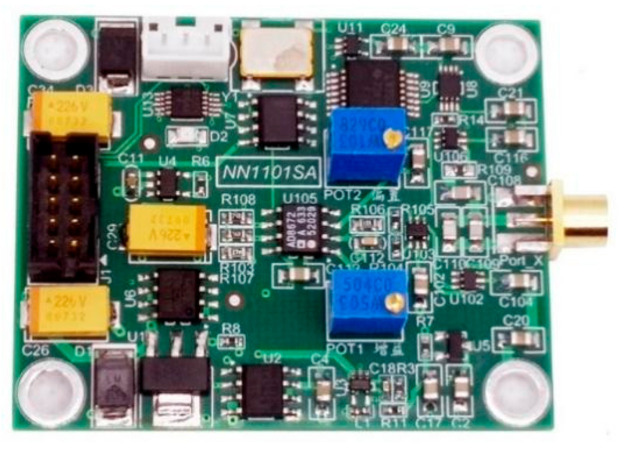
A photograph of the circuit board for single-end sensor.

A photograph of the designed single-ended eddy current micro-displacement sensor circuit with temperature compensation is shown in Figure 15. For the convenience of sensor signal acquisition, a data acquisition module was constructed with the STM32F103VCT6 microcontroller from STMicroelectronics, Geneva, Switzerland, and AD7903BRQZ analog-to-digital converter from Analog Devices Inc., Norwood, MA, USA, meeting the requirements for sampling the analog voltage range from −10 V to +10 V with 16-bit resolution.

### 5.2. Experimental Setup

**Figure 16:**
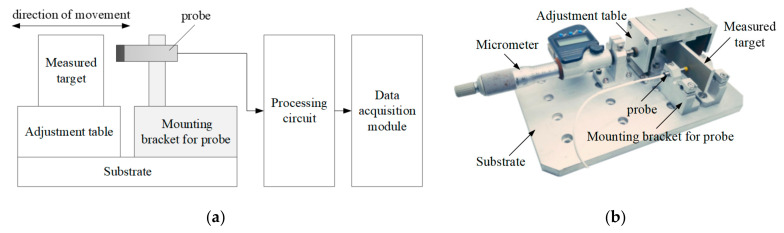
Sketch map and photograph of calibration setup: (**a**) Sketch map of the calibration setup. (**b**) Photograph of the calibration setup.

The sketch map and photograph of the calibration setup are shown in Figure 16. The distance between the measured target and the probe is adjusted by a one-dimensional precision adjustment stage. The probe is connected to the processing circuit, and the measurement results are obtained through the data acquisition module. The sensitivity of the eddy current sensor is related to the measured material [26], and in this case, aluminum (Al) is chosen as the tested material.

### 5.3. Resolution Test

**Figure 17:**
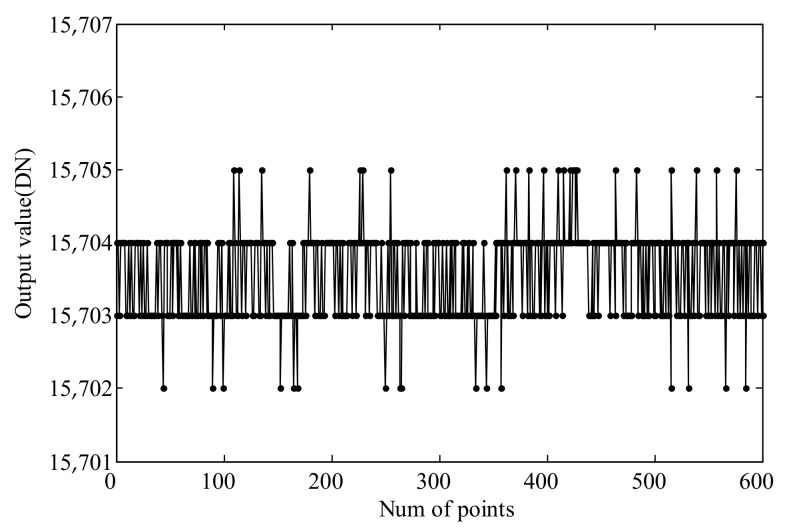
Static output of the sensor.

The data acquisition module is used to sample the static output signal of the sensor, and the sensitivity is determined based on the static output noise. As mentioned earlier, the analog input range of the data acquisition module is ±10 V, with a 16-bit digital resolution, meaning 1LSB = 0.30518 mV. The sensor range is set to 500 μm, corresponding to a full-scale output voltage range from 0 V to +10 V, resulting in a sensitivity of 20 mV/μm. The static output of the sensor obtained from the acquisition is shown in Figure 17.

It can be observed that the fluctuation of the output signal is within three units, and the sensor resolution can be calculated using the following formula:

(11) $$R=\frac{3\times 0.30518\mathrm{mV}}{20\mathrm{mV}/\mu m}\approx 46\mathrm{nm}$$

### 5.4. Sensor Calibration

**Figure 18:**
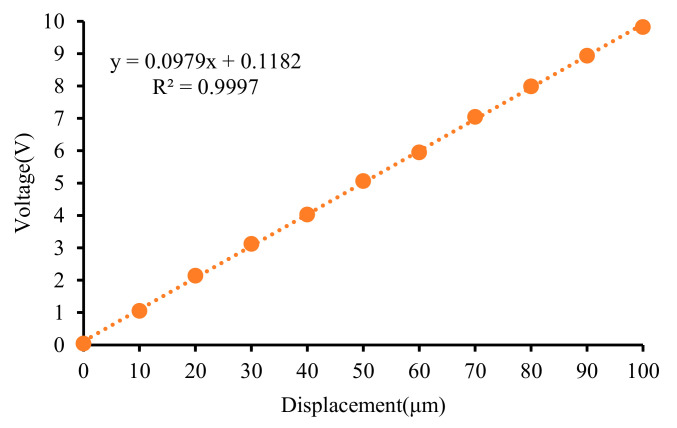
Calibration results of the sensor with measure range from 0 μm to 100 μm.

**Figure 19:**
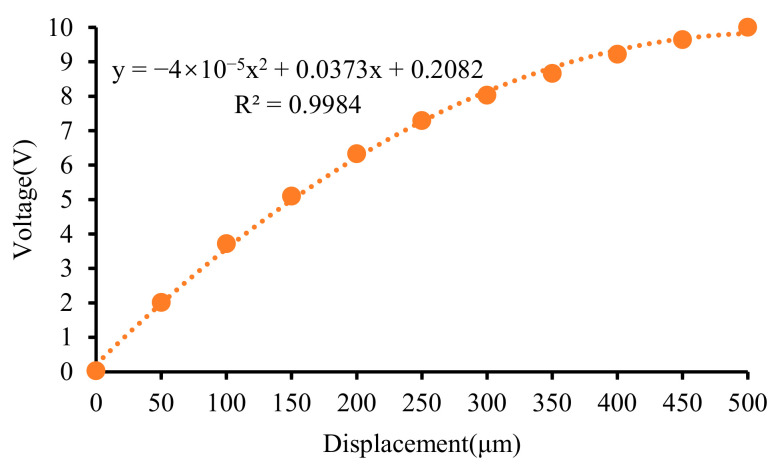
Calibration results of the sensor with measure range from 0 μm to 500 μm.

Obtaining the calibration curve of the micro-displacement sensor is crucial for its application. Setting the measurement range to 100 μm and 500 μm, respectively, the calibration results are shown in Figure 18 and Figure 19.

It can be observed that there is nonlinearity in the output of the single-ended eddy current sensor when the measurement range is relatively large.

**Figure 20:**
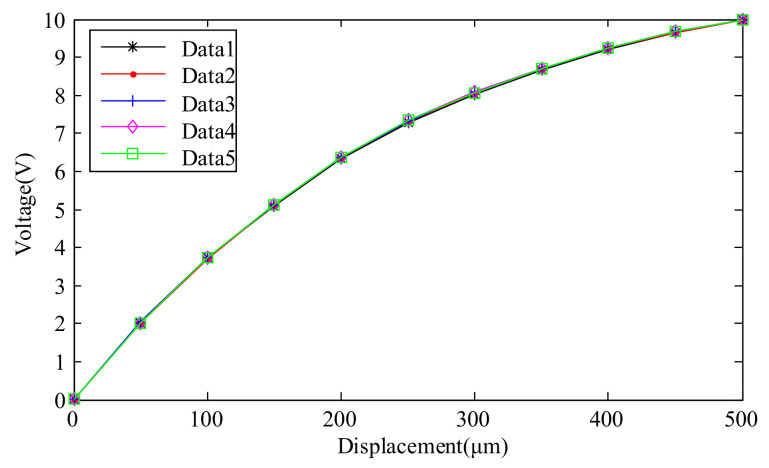
Test results of repeatability of single-ended sensor.

To test the repeatability of the sensor output signal, the sensor range was set to 500 μm, corresponding to a full-scale output voltage range from 0 V to +10 V. Adjusting the distance between the measured target and the probe in the same direction, the measurement was repeated five times. The test results are shown in Figure 20.

Taking the confidence level as 3, the repeatability *δ_R_* can be calculated using the following formula:

(12) $$\delta_{R}=\pm \frac{3\sigma_{\max}}{Y_{FS}}\times 100\%=\pm 0.70\%$$

### 5.5. Temperature Characteristics and Compensation

**Figure 21:**
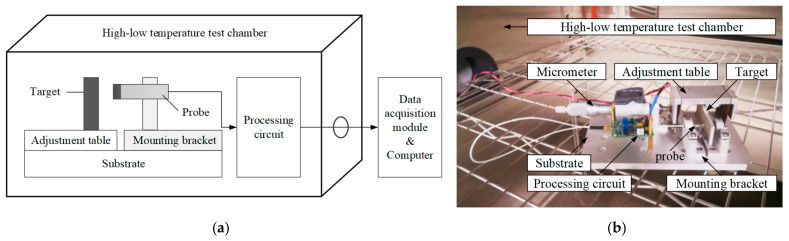
High-low temperature test schematic and physical setup: (**a**) The schematic diagram illustrates the principle of the high-low temperature test setup. It shows the arrangement of components within the test chamber and the connections between the sensor probe, the experimental setup, and the processing circuit. (**b**) The physical setup photograph provides a visual representation of the high-low temperature test apparatus. It depicts the actual equipment inside the test chamber, including the sensor probe, the experimental setup, and the processing circuit, demonstrating how the components are positioned and connected for conducting the temperature tests.

To test the temperature characteristics of the sensor, the probe, experimental setup, and processing circuit were placed in a high–low temperature test chamber with the model EGNX28-6NWL from ESPEC Environmental Equipment (Shanghai) Co., Ltd., Shanghai, China. The processing circuit was connected to an external data acquisition module via a cable, and the experimental results were collected and recorded by a computer. The schematic diagram of the high-low temperature test plan and the experimental scene are shown in Figure 21.

**Figure 22:**
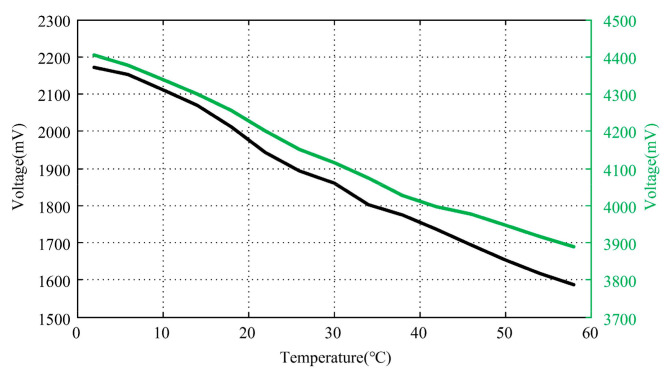
Output voltage variation curve with temperature before compensation.

At room temperature, the distance between the probe and the measured target was adjusted to achieve output voltages of approximately +1.95 V and +4.20 V, respectively. Without activating temperature compensation, the temperature was set to vary from +2 °C to +58 °C, and the curve of the output voltage variation with the temperature was measured, as shown in Figure 22.

It can be observed that the output voltage decreases with increasing temperature, and the voltage variation is slightly smaller when the measured displacement is larger. Compared with the analysis in Figure 20, it can be inferred that as the measured displacement increases, the sensitivity of the sensor gradually decreases.

**Figure 23:**
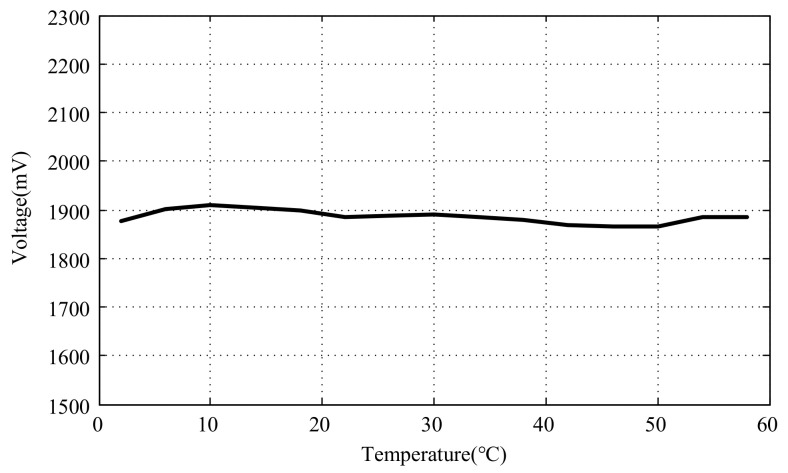
Output voltage variation curve with temperature after compensation.

To utilize the temperature compensation method proposed in this study, it is necessary to limit the sensor measurement range to a small range to ensure a certain linearity. Taking an output voltage of +1.95 V at room temperature as the basis, the output voltage should be around +1.95 V within the working temperature range after activating the temperature compensation. The curve of the output voltage variation with the temperature after compensation is shown in Figure 23.

It can be observed that before compensation, the change in output voltage is 586 mV, while after compensation, the maximum voltage fluctuation is reduced to 44 mV, resulting in a significant decrease in output fluctuation.

## 6. Conclusions

Given the advantages of eddy current sensors, including their large measurement linear range, simple structure, low cost, high sensitivity, wide frequency band, immunity to oil contamination, and strong anti-interference ability, a comprehensive study was conducted on a single-ended eddy current sensor with temperature compensation.

When the distance between the probe and the measured target changes, the amplitude and phase of the sensor probe’s output signal will change accordingly. The processing circuit of the single-ended eddy current sensor converts the change in output signal into a corresponding pulse width and extracts the phase difference between it and the reference pulse formed by the exciting signal. This phase difference is then transformed into an analog voltage through a low-pass filter, and the final measurement result is obtained after amplification and temperature compensation. Finally, experiments were conducted using the established experimental setup.

The results show that nonlinearity exists in the output of a single-ended eddy current sensor when the measurement range is relatively large. When the range is set to 500 μm, the sensor resolution can reach 46 nm, with a repeatability error of ±0.70% FR. In the temperature range from +2 °C to +58 °C, temperature compensation using a digital potentiometer with an embedded temperature sensor can reduce the voltage fluctuation of the sensor output from 586 mV to 44 mV. In practical applications, the selection of range, accuracy requirements, and target material should be balanced.

## Data Availability

The original contributions presented in the study are included in the article. Further inquiries can be directed to the corresponding authors.

## References

[1] Wang K., Ou Y., Zeng S., Feng H., Wu J. (2020). Measurement of ball nut profile using laser sensors. Opt. Precis. Eng..

[2] Xu L., Sun S., Cao Z., Yang W. (2015). Performance analysis of a digital capacitance measuring circuit. Rev. Sci. Instrum..

[3] Wang W., Yang H., Zhang M., Chen Z., Shi G., Lu K., Xiang K., Ju B. (2018). A novel method for the micro-clearance measurement of a precision spherical joint based on a spherical differential capacitive sensor. Sensors.

[4] Ma T., Yang S., Xu Y., Liu D., Hou J., Liu Y. (2022). Analysis and Correction of Measurement Error of Spherical Capacitive Sensor Caused by Assembly Error of the Inner Frame in the Aeronautical Optoelectronic Pod. Sensors.

[5] Yang S., Xu Y., Xu Y., Ma T., Wang H., Hou J., Liu D., Shen H. (2022). A Novel Method for Detecting the Two-Degrees-of-Freedom Angular Displacement of a Spherical Pair, Based on a Capacitive Sensor. Sensors.

[6] Kumar A.A., George B. (2019). A noncontact angle sensor based on eddy current technique. IEEE Trans. Instrum. Meas..

[7] Harms J., Kern T.A. (2021). Theory and Modeling of Eddy Current Type Inductive Conductivity Sensors. Eng. Proc..

[8] Ali K.B., Abdalla A.N., Rifai D., Faraj M.A. (2017). Review on system development in eddy current testing and technique for defect classification and characterization. IET Circuits Devices Syst..

[9] Ma K., Yang Q., Zhang J., Dang X., Hu P. (2022). A New 2D Displacement Measurement Method Based on an Eddy Current Sensor and Absolute Encoding. Machines.

[10] Dziczkowski L., Tytko G. (2023). Evaluation of the Properties of Eddy Current Sensors Based on Their Equivalent Parameters. Sensors.

[11] Farag H.E., Toyserkani E., Khamesee M.B. (2022). Non-Destructive Testing Using Eddy Current Sensors for Defect Detection in Additively Manufactured Titanium and Stainless-Steel Parts. Sensors.

[12] Xia Z., Huang R., Chen Z., Yu K., Zhang Z., Salas-Avila J.R., Yin W. (2022). Eddy Current Measurement for Planar Structures. Sensors.

[13] Liao C., Yi Y., Chen T., Cai C., Deng Z., Song X., Lv C. (2022). Detecting Broken Strands in Transmission Lines Based on Pulsed Eddy Current. Metals.

[14] Chandran P., Thiery F., Odelius J., Lind H., Rantatalo M. (2022). Unsupervised Machine Learning for Missing Clamp Detection from an In-Service Train Using Differential Eddy Current Sensor. Sustainability.

[15] Harms J., Kern T.A. (2021). Design and Potential Analysis of an Eddy Current Sensor for Inductive Conductivity Measurement in Fluids. Eng. Proc..

[16] Xie Y., Li J., Tao Y., Wang S., Yin W., Xu L. (2020). Edge Effect Analysis and Edge Defect Detection of Titanium Alloy Based on Eddy Current Testing. Appl. Sci..

[17] Li W., Ye Y., Zhang K., Feng Z. (2017). A thickness measurement system for metal films based on eddy-current method with phase detection. IEEE Trans. Ind. Electron..

[18] Wang Z., Zhang B., Li X., Zhang S. (2020). Application of fast steering mirror in image motion compensation. Chin. Opt..

[19] Huang P., Yang X., Xiu J., Li J., Li Y. (2020). Reduced-order active disturbance rejection control of fast steering mirror driven by VCA. Opt. Precis. Eng..

[20] Ma T., Han Y., Xu Y., Dai P., Shen H., Liu Y. (2023). Wide Temperature Range and Low Temperature Drift Eddy Current Displacement Sensor Using Digital Correlation Demodulation. Sensors.

[21] Lei B., Yi P., Li Y., Xiang J. (2018). A temperature drift compensation method for pulsed eddy current technology. Sensors.

[22] Li Q., Ding F. (2005). Novel displacement eddy current sensor with temperature compensation for electrohydraulic valves. Sens. Actuators A Phys..

[23] Wang S.-C., Xie B.-R., Huang S.-M. (2022). Design and Analysis of Small Size Eddy Current Displacement Sensor. Sensors.

[24] Song M., Li M., Xiao S., Ren J. (2023). Research on the Influence of Geometric Structure Parameters of Eddy Current Testing Probe on Sensor Resolution. Sensors.

[25] DS3501: High-Voltage, NV, I2C POT with Temp Sensor and Lookup Table [EB/OL]. https://www.analog.com/media/cn/technical-documentation/data-sheets/DS3501_cn.pdf.

[26] Bertacchini A., Lasagni M., Sereni G. (2020). Effects of the Target on the Performance of an Ultra-Low Power Eddy Current Displacement Sensor for Industrial Applications. Electronics.

